# Popliteal vein aneurysm: report of two cases

**DOI:** 10.1590/1677-5449.009717

**Published:** 2018

**Authors:** Marcio Miyamotto, Marina de Lorenzo Costa, Victor Hugo Granella, Bruna Zimmerman Angelo, Danielle Corrêa de Andrade, Cintia Lopes Raymundo, Ricardo César Rocha Moreira

**Affiliations:** 1 Pontifícia Universidade Católica do Paraná – PUC-PR, Hospital Universitário Cajuru – HUC, Serviço de Cirurgia Vascular e Endovascular, Curitiba, PR, Brasil.; 2 Instituto VESSEL de Aperfeiçoamento Endovascular de Curitiba, Curitiba, PR, Brasil.; 3 Hospital Nossa Senhora das Graças – HNSG, Serviço de Cirurgia Vascular e Endovascular Elias Abrão, Curitiba, PR, Brasil.; 4 Pontifícia Universidade Católica do Paraná – PUC-PR, Hospital Universitário Cajuru – HUC, Liga Acadêmica de Medicina Vascular – LAMEV, Curitiba, PR, Brasil.

**Keywords:** aneurysm, popliteal vein, deep vein thrombosis

## Abstract

Venous aneurysms are rare and often diagnosed incidentally. Popliteal vein aneurysms are the most common type of venous aneurysms and have a strong association with the occurrence of deep vein thrombosis and recurrent pulmonary embolism. This article reports two cases of popliteal vein aneurysms associated with deep vein thrombosis.

## INTRODUCTION

 Venous aneurysms were first described by Sir William Osler in 1913 and can theoretically involve any venous segment in the body. 1 Popliteal vein aneurysms (PVA) are the most common venous aneurysms and were first described in 1968 by May and Nissl. 2 A review conducted in 2006 by Bergqvist et al. 3 identified 147 cases of this pathology. The objective of this article is to describe two cases of PVA and the management chosen for each. 

## CASE REPORTS

### Case 1

**Figure 1:**
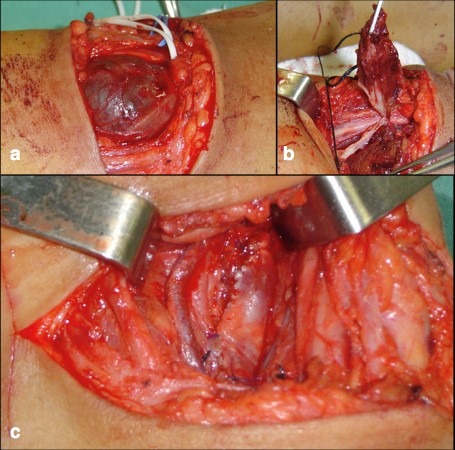
Posterior access (a), aneurysmectomy (b) and lateral venorrhaphy (c).

 A 15-year-old male patient presented complaining of pain and localized swelling in the right popliteal fossa with onset 1 month previously. He reported a prior history of vascular malformation in the posterior aspect of the right thigh, which had never been diagnosed definitively. Physical examination revealed a firm mass in the right popliteal fossa associated with diffuse increase of volume in the posterior region of the distal right thigh. Doppler ultrasonography was used to diagnose a voluminous right PVA. In view of the risk of thrombosis and the patient’s symptoms, the treatment chosen was open surgical venous aneurysmectomy followed by primary reconstruction of the popliteal vein ( Figure 1 ). The patient was prescribed treatment with anticoagulants for 3 months and instructed to attend for clinical follow-up. 

### Case 2

**Figure 2:**
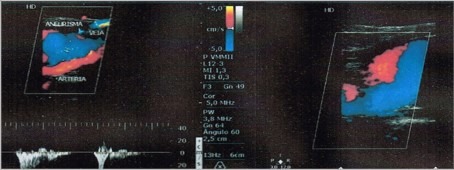
Doppler ultrasonography showing the popliteal vein aneurysm.

 A 66-year-old female patient presented with pain and edema of the left lower limb as far as the top of the thigh. The only risk factor for thrombosis that she reported when interviewed was immobilization lasting a few hours. Doppler ultrasonography showed venous thrombosis of the left gastrocnemius and fibular veins and a left PVA ( Figure 2 ). She was managed with systemic anticoagulation, taking Rivaroxaban for 6 months, and instructed to care for the site with localized heat, elevation, and compression therapy with medium pressure elastic stockings. 

## DISCUSSION

 Aneurysms of the popliteal vein are rare, although their prevalence has not been established, since there are no studies employing screening. Descriptions in the literature report prevalence rates below 0.5%. 4 ^,^ 5 There is no information on the ratio of symptomatic to asymptomatic cases. 3

 In the first published reports of PVA, the most common clinical presentation – in up to 80% of cases – was venous thromboembolism (VTE). Nowadays, with greater availability of noninvasive imaging methods such as Doppler ultrasonography, early diagnosis of venous aneurysms in the pre-thrombotic phase or as an incidental finding in asymptomatic patients has become much more common. 6 In the series reported by Sessa et al., 7 Doppler ultrasonography was used to diagnose all 25 cases of PVA. Pain in the knee area or a mass in the popliteal fossa were present in 38% of cases in a study published by Bergqvist et al. 3 Donaldson et al. 6 described a sample of 21 patients, 52% of whom had symptoms of chronic venous insufficiency and 14% of pulmonary embolism, while 19% were diagnosed with deep venous thrombosis and 29% had incidental findings. 

 Approximately two thirds of PVAs are saccular, a majority are located in the left lower limb, 8 ^,^ 9 and in 25% of cases they occur bilaterally, underscoring the importance of examining both legs with Doppler ultrasonography. The higher prevalence of PVA on the left side led to the suggestion that there is an association with compression of the left common iliac vein by the right common iliac artery, which was described in three asymptomatic patients diagnosed with imaging exams. 10 However, in the series described by Donaldson et al., 6 12 patients with PVA underwent angiotomography or magnetic resonance angiography of the iliac-cava territory and there was no evidence of venous compression in the area. 

 Popliteal vein aneurysm should be considered a differential diagnosis possibility in patients with increased volume in the region of the popliteal fossa and in young patients with VTE in whom screening for thrombophilia is negative. 

 The traditional treatment of choice is surgical repair for patients with aneurysms larger than 3 cm, recurrent thrombosis or pulmonary embolism after anticoagulation, and high risk for venous thrombosis. In a study published in 2013 by Maldonado-Fernandez et al., 9 pulmonary embolism followed by death was observed in five patients treated with systemic anticoagulation alone. The literature contains conflicting data on recurrence of VTE in patients treated with anticoagulation alone, with rates ranging from 0 to 80%. 6 ^,^ 11 Recurrent VTE is described little and there are no published cases of death from pulmonary embolism in patients treated with surgical repair. 7 ^,^ 9 ^,^ 11

 Several surgical techniques are available to treat popliteal vein aneurysms and the choice depends on the anatomy of the aneurysm. Posterior access is most common, followed by resection of the aneurysm sac and lateral venorrhaphy to reconstruct the vein. 7 It may be necessary to use patch venoplasty for vascular reconstruction or even an autologous bypass graft. Ligature of the popliteal vein with aneurysmectomy is not a recommended technique, considering the long term sequelae related to venous insufficiency and edema in the lower limbs. 12 ^,^ 13 The anticoagulation regimen after surgical treatment is not yet well-established, but anticoagulation periods of from 6 to 8 weeks are suggested as reasonable to allow endothelization of the area repaired. 3 Some authors recommend at least 3 months of anticoagulation. 7 ^,^ 14

 In conclusion, popliteal vein aneurysms are rare, but can potentially be fatal, because of the risk of pulmonary embolism. Doppler ultrasonography is the diagnostic method of choice. Although there is no consensus on treatment of patients with PVA, the individual level of risk of recurrence of venous thrombosis should be considered, and surgery is the most recommended option in these cases. 
