# Supplementary material for: Popliteal vein aneurysm: report of two cases
Source: J Vasc Bras. 2018 Apr-Jun;17(2):170–3. doi: 10.1590/1677-5449.009717 (PMC6205701; doi:10.1590/1677-5449.009717)
Supplement: Translation

# Translation: Aneurisma de veia poplítea: relato de dois casos

**Authors:** Marcio Miyamotto, Marina de Lorenzo Costa, Victor Hugo Granella, Bruna Zimmerman Angelo, Danielle Corrêa de Andrade, Cintia Lopes Raymundo, Ricardo César Rocha Moreira

**Affiliations:** 1 Pontifícia Universidade Católica do Paraná – PUC-PR, Hospital Universitário Cajuru – HUC, Serviço de Cirurgia Vascular e Endovascular, Curitiba, PR, Brasil.; 2 Instituto VESSEL de Aperfeiçoamento Endovascular de Curitiba, Curitiba, PR, Brasil.; 3 Hospital Nossa Senhora das Graças – HNSG, Serviço de Cirurgia Vascular e Endovascular Elias Abrão, Curitiba, PR, Brasil.; 4 Pontifícia Universidade Católica do Paraná – PUC-PR, Hospital Universitário Cajuru – HUC, Liga Acadêmica de Medicina Vascular – LAMEV, Curitiba, PR, Brasil.

**Keywords:** aneurisma, veia poplítea, trombose venosa profunda

## Abstract

Os aneurismas venosos são raros, sendo na maioria das vezes diagnosticados de forma incidental. Os aneurismas de veia poplítea são os mais comuns entre os aneurismas venosos e apresentam uma forte associação com a ocorrência de trombose venosa profunda e embolia pulmonar recorrente. O presente estudo descreve dois casos de aneurisma de veia poplítea associados a trombose venosa profunda.

## INTRODUÇÃO

 Os aneurismas venosos foram descritos inicialmente por Sir William Osler no ano de 1913 e podem acometer teoricamente qualquer segmento venoso do organismo 1 . O aneurisma da veia poplítea (AVP) é o aneurisma venoso mais comum, sendo descrito inicialmente em 1968 por May e Nissl 2 . Em uma revisão realizada em 2006 por Bergqvist et al. 3 , foram identificados 147 casos dessa doença. O objetivo deste artigo é descrever dois casos de AVP e o manejo escolhido para cada um. 

## RELATO DOS CASOS

### Caso 1

 Paciente com 15 anos de idade do sexo masculino apresentava queixa de dor associada a um aumento de volume localizado em fossa poplítea direita com início há um mês. Relatou história prévia de malformação vascular em região posterior de coxa direita, porém sem diagnóstico definido. Ao exame físico, apresentava uma massa de consistência firme na região de fossa poplítea direita associada a aumento de volume difuso em região posterior distal da coxa direita. Foi realizado eco-Doppler, que diagnosticou volumoso AVP à direita. Devido ao risco de trombose e aos sintomas exacerbados do paciente, optou-se por abordagem cirúrgica com aneurismectomia venosa e reconstrução primária da veia poplítea ( Figura 1 ). Paciente foi orientado a manter tratamento com anticoagulantes por 3 meses e buscar acompanhamento clínico. 

**Figura 1:** Acesso posterior (a), anerismectomia (b) e venorrafia lateral (c).

### Caso 2

 Paciente com 66 anos de idade do sexo feminino apresentava quadro de dor e edema em membro inferior esquerdo desde a raiz da coxa. Relatava história de imobilização de poucas horas como único fator de risco detectável para trombose na anamnese. O eco-Doppler mostrou, além da trombose venosa de veias gastrocnêmias e fibulares à esquerda, presença de AVP ( Figura 2 ). A paciente foi manejada com anticoagulação sistêmica utilizando rivaroxabana por 6 meses, além de cuidados locais como calor local e elevação dos membros e terapia compressiva com meia elástica de média compressão. 

**Figura 2:** Eco-Doppler mostrando aneurisma de veia poplítea.

## DISCUSSÃO

 Os aneurismas vasculares poplíteos são raros e de prevalência não estabelecida, uma vez que não existem estudos de rastreamento. Descrições na literatura relatam prevalências abaixo de 0,5% 4 ^,^ 5 . Não há informações sobre a relação entre casos sintomáticos e assintomáticos 3 . 

 Nos primeiros relatos de AVP publicados, a apresentação clínica mais comum – em até 80% – era o tromboembolismo venoso (TEV). Atualmente, com a maior disponibilidade de métodos de imagem pouco invasivo como o eco-Doppler, o diagnóstico precoce de aneurismas venosos na fase pré-trombótica ou como achado incidental em pacientes assintomáticos tornou-se cada vez mais frequente 6 . Na série relatada por Sessa et al. 7 , o eco-Doppler foi o exame utilizado para o diagnóstico em todos os 25 casos de AVP. Dor na região do joelho ou presença de massa na fossa poplítea foram relatadas em 38% dos casos no estudo de Bergqvist et al. 3 . Donaldson et al. 6 descreveram uma amostra de 21 pacientes, na qual 52% possuíam sintomas de insuficiência venosa crônica e 14% de embolia pulmonar, 19% foram diagnosticados com trombose venosa profunda e 29% tiveram achados incidentais. 

 Aproximadamente dois terços dos AVP são saculares, a maioria localiza-se no membro inferior esquerdo 8 ^,^ 9 e cerca de 25% ocorrem de forma bilateral, ressaltando a importância de examinar ambas as pernas ao eco-Doppler. Devido à maior prevalência do AVP do lado esquerdo, foi proposta uma associação com a compressão da veia ilíaca comum esquerda pela artéria ilíaca comum direita, descrita em três pacientes assintomáticos submetidos a exames de imagem 10 . Porém, na série publicada por Donaldson et al. 6 , 12 pacientes com AVP foram submetidos a angiotomografia ou a angiorressonância do território ilíaco-cava e não houve evidência de compressão venosa nesse território. 

 O AVP deve ser considerado como um dos diagnósticos diferenciais em pacientes com aumento de volume na região da fossa poplítea e em pacientes jovens com TEV em que a triagem para trombofilia é negativa. O exame inicial de escolha é a ultrassonografia com Doppler venoso, por permitir a exclusão diagnóstica e a avaliação de potenciais diagnósticos diferenciais 3 . 

 O tratamento de escolha tem sido tradicionalmente o reparo cirúrgico em pacientes com aneurismas maiores que 3 cm, recorrência de trombose ou embolia pulmonar após anticoagulação e alto risco para trombose venosa. A ocorrência de embolia pulmonar e óbito foi observada em cinco pacientes tratados com anticoagulação sistêmica isolada no estudo de Maldonado-Fernandez et al. 9 . A literatura possui dados divergentes quanto à recorrência de TEV em pacientes tratados apenas com anticoagulação, variando de 0 a 80% 6 ^,^ 11 . A recorrência de TEV é pouco descrita e não há casos publicados de morte por embolia pulmonar em pacientes submetidos a correção cirúrgica 7 ^,^ 9 ^,^ 11 . 

 Existem várias técnicas cirúrgicas disponíveis para o tratamento do aneurisma de veia poplítea e sua escolha varia de acordo com a anatomia do aneurisma. O acesso posterior é o mais utilizado, seguido de ressecção do saco aneurismático e venorrafia lateral para reconstrução venosa 7 . Pode haver a necessidade de reconstrução vascular através de venoplastia com remendo ou mesmo de confecção de uma ponte com enxerto autógeno. A ligadura da veia poplítea com aneurismectomia não é uma técnica recomendada, considerando as sequelas a longo prazo relacionadas a insuficiência venosa e edema de membros inferiores 12 ^,^ 13 . O regime da anticoagulação após o tratamento cirúrgico ainda não é bem estabelecido, mas sugere-se um período de 6 a 8 semanas de anticoagulação como razoável para a endotelização da área reconstruída 3 . Alguns autores recomendam pelo menos 3 meses de anticoagulação 7 ^,^ 14 . 

 Em conclusão, os aneurismas venosos poplíteos são raros, mas potencialmente fatais, pelo risco de embolia pulmonar. O eco-Doppler é o método diagnóstico de escolha. Apesar de não haver consenso quanto ao tratamento dos pacientes com AVP, deve-se levar em conta o risco individual de recorrência de trombose venosa, sendo a cirurgia mais indicada nesses casos.
